# Aptamer-Based Planar Electric Double-Layer Field-Effect Transistor: A Novel Approach for Sensitive Troponin I Sensing

**DOI:** 10.3390/bios15050285

**Published:** 2025-04-30

**Authors:** Sheng-Chun Hung, Yi-Hua Lee

**Affiliations:** Department of Electrical Engineering, Feng Chia University, Taichung City 407102, Taiwan; m1310904@o365.fcu.edu.tw

**Keywords:** aptamer-based sensing, planar electric double-layer, field-effect transistor, troponin I detection, debye length effects, point-of-care diagnostics

## Abstract

This study introduces a cutting-edge, aptamer-based, planar electric, double-layer field-effect transistor (FET) system that offers both high sensitivity and specificity for the detection of troponin I (TnI). The proposed sensing platform leverages the signal amplification capabilities of FETs alongside the unique attributes of a planar electric double-layer design to address the limitations inherent in traditional ion-sensitive detectors, which are impacted by Debye length effects. By integrating TnI-specific aptamers, the system markedly enhances molecular recognition and transduction efficiency, achieving an impressive detection limit of 0.0001467 decade. Furthermore, the sensor demonstrates a strong exponential linear response across a clinically relevant concentration range of 1 ng/mL to 100 ng/mL. This innovative approach underscores the potential of electric double-layer FET systems to advance biomarker detection technologies for medical diagnostics and point-of-care applications.

## 1. Introduction

Cardiovascular diseases (CVDs) rank among the leading causes of death globally. Recent statistics reveal that fatalities attributed to CVD have increased from approximately 12.1 million in 1990 to around 20.5 million in 2021, marking a substantial rise of 60%. In 2019 alone, CVD resulted in approximately 17.9 million deaths, accounting for 32% of total global mortality. The primary contributor is ischemic heart disease (IHD), with acute myocardial infarction (AMI) being the most prevalent cause of death within acute coronary syndrome [1,2,3,4,5].

In Taiwan, heart disease consistently ranks among the top three causes of death, highlighting the particularly high mortality rate associated with AMI and underscoring the critical importance of early detection. AMI occurs when blood flow to the myocardium is abruptly diminished or entirely obstructed, resulting in myocardial necrosis and subsequent cardiac events. Patients typically exhibit symptoms such as chest pain, fatigue, sweating (diaphoresis), nausea, vomiting, and, occasionally, loss of consciousness [6,7,8].

The primary causes of AMI include arterial embolism and thrombosis. Due to the irreversible myocardial damage caused by hypoxia, AMI may lead to severe complications, including arrhythmias, embolisms, and aneurysms. High-risk groups consist of individuals with diabetes, hypertension, or pre-existing heart conditions, as well as elderly patients aged over 75 years.

Based on etiology, AMI can be classified into five types: Type I involves spontaneous ischemic infarction due to the erosion or rupture of coronary artery plaques; Type II results from an imbalance between oxygen supply and demand due to embolism, arrhythmias, or abnormal blood pressure; Type III refers to sudden cardiac death occurring before biomarkers are detected; Type IV is associated with coronary intervention or stent thrombosis; and Type V arises from coronary artery bypass graft surgery [9,10]. Timely diagnosis and treatment within the initial hours following an AMI are essential for significantly reducing adverse outcomes. Current diagnostic methods encompass physical examinations, electrocardiograms (ECGs) [11], imaging studies, and biomarker analyses [12]. Given that 85% of myocardial injury occurs within the first two hours after the onset of AMI, early diagnosis and intervention within six hours are vital for enhancing survival rates. Key cardiac biomarkers, such as creatine kinase–MB (CK-MB) and cardiac troponin (cTn), are crucial for diagnosing myocardial necrosis [13,14]. While ECGs remain the cornerstone for diagnosing AMI, only about 57% of patients display abnormal ECG results. Consequently, measuring specific cardiac biomarker concentrations in the blood has emerged as a vital strategy for identifying high-risk AMI patients. This method not only facilitates the effective allocation of healthcare resources but also helps reduce medical costs. The early detection of these biomarkers not only aids in preventing complications but also significantly decreases the long-term risk of symptom recurrence.

Current biomarker detection technologies, such as enzyme-linked immunosorbent assays (ELISA) [15], fluorescence detection [16,17], and surface plasmon resonance (SPR) [18,19], are known for their high sensitivity. However, these methods often require laboratory environments, specialized equipment, and longer turnaround times. Given that AMI patients can deteriorate swiftly, timely diagnosis is paramount. The American Heart Association recommends that the interval from sample collection to result reporting be kept under 60 min, ideally within 30 min. Additionally, the National Academy of Clinical Biochemistry notes that within the first 6 to 9 h following the onset of AMI, specific biomarker concentrations in the blood typically range from picomolar (pM) to nanomolar (nM) [20]. Aptamers have emerged as powerful alternatives to antibodies for detecting myocardial infarction (MI), with troponin I (TnI) serving as a highly specific marker for myocardial injury; its detection is crucial for early MI diagnosis.

Aptamers are short, single-stranded oligonucleotides generated through the SELEX (Systematic Evolution of Ligands by Exponential Enrichment) process, which enables the selection of sequences with high affinity and specificity for a wide range of target molecules, including cardiac troponin I (cTnI) [21]. These nucleic acids adopt unique three-dimensional conformations that facilitate specific binding to their targets via non-covalent interactions such as hydrogen bonding, electrostatic forces, and hydrophobic interactions. Unlike antibodies, aptamers can be rationally designed and chemically synthesized to minimize cross-reactivity, a feature that is especially advantageous in complex biological matrices like blood. The exceptional binding affinity of aptamers—often comparable to or exceeding that of antibodies—is critical for detecting low concentrations of cardiac biomarkers such as cTnI, which gradually increase in the bloodstream following myocardial injury [22]. High-sensitivity detection is thus essential for early diagnosis and timely therapeutic intervention. Furthermore, aptamers possess several practical advantages over antibodies, including superior chemical stability, lower batch-to-batch variation, reduced immunogenicity, and ease of functional modification. These characteristics make aptamer-based biosensors highly suitable for clinical diagnostics, providing an accurate, selective, and sensitive detection of cTnI with a reduced risk of false positives.

Aptamers also offer greater stability than antibodies. They maintain chemical stability at room temperature and can withstand a wider range of environmental conditions without requiring refrigeration or special handling to preserve their activity. This high level of stability makes them particularly well-suited for portable diagnostic devices that must operate reliably in diverse environments. Moreover, their durability extends their shelf life and reduces storage costs, making them an economically viable solution for long-term diagnostic applications [23,24].

The production method for aptamers presents another significant advantage. In contrast to antibodies, which require complex and costly production processes, aptamers are generated through relatively straightforward and cost-effective in vitro synthesis techniques. The SELEX method provides scalability and economic feasibility, which is especially advantageous for large-scale manufacturing—an essential factor for developing wearable or portable diagnostic devices.

This study introduces a novel technology that employs an aptamer-based, planar electric double-layer FET for the detection of TnI. This method harnesses the remarkable specificity of aptamers for TnI and utilizes the adjustable spacing characteristics of planar electric double layers to effectively address the limitations of Nernst’s equation in electrochemistry. Additionally, it enhances electrical signals through the use of transistors, offering a state-of-the-art solution that fulfills the clinical requirements.

Field-effect transistors are increasingly acknowledged for their significant advantages as biochemical sensors. Firstly, FET sensors exhibit remarkable sensitivity, being capable of detecting extremely low concentrations of biomolecules—down to 10–12 g/mL—while maintaining a robust performance even in high-salinity environments. Secondly, these sensors can be precisely designed to selectively identify target biomarkers within complex biological samples [25,26].

Furthermore, FET sensors provide rapid detection capabilities, with analyses typically completed within minutes, which is a considerable advantage in clinical contexts. Many contemporary FET biosensors are designed as user-friendly portable devices that do not require specialized technical expertise, allowing a broad user base to easily obtain testing results. Most FET sensors also do not necessitate the labeling or modification of samples prior to testing, thus simplifying the sample processing and reducing overall testing costs.

In summary, the proposed aptamer-based planar electric double-layer field-effect transistor technology not only overcomes the limitations of traditional diagnostic methods but also enables the rapid, highly sensitive, and specific detection of biomarkers such as TnI. This advancement facilitates the early diagnosis and treatment of acute myocardial infarction, ultimately enhancing patient outcomes.

## 2. Materials and Methods

### 2.1. Materials and Chemicals

Hydrochloric acid (HCl), Tris(2-carboxyethyl) phosphine hydrochloride (TCEP), sodium dodecyl sulfate (SDS), and 10× phosphate-buffered saline (10× PBS) were acquired from Sigma Aldrich. A 0.02× PBS solution was freshly prepared by diluting 10× PBS with deionized water and was utilized in all experiments as both the assay buffer and the washing solution. The ionic strength of 1× PBS is approximately 150 mM, which means that an ionic concentration of 0.02× PBS is estimated to be about 3 mM. This concentration was consistently employed in all electrochemical and fluorescence measurements and was also used to calculate the Debye length that was relevant to the sensing environment.

### 2.2. Surface Functionalization

**Figure 1:**
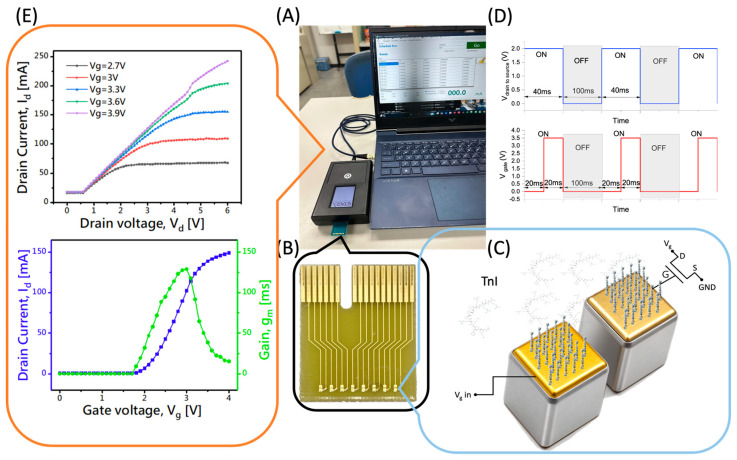
(**A**) The system setup comprises a sensor chip, a reader, and a user interface presented on a laptop computer. (**B**) The sensor chip features eight individual sensors arranged in a 1 × 8 configuration. (**C**) A schematic representation of the extended-gate FET with immobilized Tro4 aptamers is depicted. (**D**) A square-wave voltage signal is applied to the sensor electrode, with gate and drain voltages alternating at 20 ms on and 20 ms off intervals, followed by a 100 ms off-state period. (**E**) Electrical characterization of the MOSFET: the upper plot presents the output characteristics, illustrating the drain current (I_d_) as a function of drain voltage (V_d_) under various gate voltages (Vg), while the lower plot shows the transfer characteristics, including the drain current (I_d_) as a function of gate voltage (V_g_), and the corresponding transconductance (g_m_) measured at a fixed drain voltage of Vd = 2 V.

In this study, we employed an Extended-Gate Field-Effect Transistor (EGFET) architecture for the fabrication of a biosensor. The EGFET is a modified variant of the conventional metal–oxide–semiconductor field-effect transistor (MOSFET), in which the gate terminal is physically separated from the transistor body and connected to an external sensing electrode. This arrangement allows the sensing surface to be exposed to aqueous environments while protecting the semiconductor from chemical degradation, making it highly suitable for biosensing applications. The extended gate and the sensor chip used in the EGFET structure are shown in Figure 1B. The sensing electrode was sterilized through autoclaving to prepare the EGFET sensor chip. The sterilization conditions were set to 121 °C at a 1.2 kg/cm^2^ pressure for 15 min. Following sterilization, the surface was rinsed sequentially with ethanol and deionized (DI) water, then dried using a nitrogen gun. This was succeeded by gate surface modifications to facilitate the immobilization of aptamers. The extended gate structure allowed for chemical functionalization of the sensing region without interfering with the internal electronics of the transistor, thereby enhancing the stability and reproducibility of biological measurements. A 3 min oxygen plasma (O_2_ plasma) treatment was administered to activate the gold surface by generating reactive sites, including hydroxyl and peroxide groups, as well as dangling bonds. This procedure, as documented in prior studies [27,28], serves to enhance surface reactivity effectively.

Following this, the chip was immersed in an acid-wash solution consisting of deionized (DI) water and hydrochloric acid (HCl) in a 4:10 ratio for 3 min. The chip was then thoroughly rinsed with DI water and dried with nitrogen gas. This step further refined the surface functional groups, optimizing the gold surface for improved aptamer attachment and ensuring robust immobilization for the subsequent sensor development.

The sensing electrode was functionalized with the Tro4 aptamer, a single-stranded DNA sequence specifically selected for its high affinity and specificity for cardiac troponin I (cTnI). The aptamers utilized in this study were synthesized by Genomics Bioscience Co., Ltd. (New Taipei City, Taiwan) and possess the following nucleotide sequence:

5′-CGTGCAGTACGCCAACCTTTCTCATGCGCTGCCCCTCTTA-3′.

To enable site-specific immobilization and fluorescence-based signal verification, the aptamer was modified with a disulfide bond (S–S) at the 5′ end, facilitating its anchoring to the gold electrode surface through thiol–gold chemistry. Additionally, an FAM (6-carboxyfluorescein) fluorophore was attached to the 3′ end. This surface modification strategy ensures the stable and oriented immobilization of the aptamer, allowing for the selective and efficient detection of TnI in the biosensing assay.

For the preparation of aptamers, they were rehydrated in phosphate-buffered saline (PBS) to restore their biological activity. The solution was vortexed for a duration of 5 s to ensure thorough mixing. Subsequently, it was stored at −20 °C to prevent degradation and maintain stability.

In the aptamer immobilization process, the aptamer was first combined with sodium dodecyl sulfate (SDS) in a 1:9 ratio and vortexed for 5 s. The SDS disrupts the aptamer’s secondary and tertiary structures, resulting in its linearization. This denaturation step enhances the precision of molecular weight assessments during electrophoretic analysis.

An equal volume of tris(2-carboxyethyl)phosphine (TCEP) was then added to the mixture, followed by a 5 s vortex. TCEP specifically reduces disulfide bonds, converting them into free thiol (−SH) groups. This reduction process is essential for examining protein and aptamer structures, as disulfide bonds play a significant role in determining folding and stability.

The solution was heated to 90 °C for 1 min and subsequently vortexed for 5 s to maximize the reduction efficiency of TCEP. This process facilitated the complete reduction of disulfide bonds into free thiol groups, thereby improving the structural integrity and functional performance of the aptamer. The denaturation step ensured the aptamer adopted a fully linearized conformation, optimizing its interaction potential.

Following this preparation, 20 μL of the solution was carefully dispensed onto the sensor chip. After incubation at 24 °C for 24 h, unbound aptamer probes were removed via rinsing the sensor chip with a 0.02× PBS buffer solution. The fluorescence intensity of the immobilized aptamers was analyzed using a fluorescence microscope (LEICA DM2500 LED, Major Instruments Co., Ltd., Taipei, Taiwan) and quantified with ImageJ analysis software (version 1.53t, National Institutes of Health, Bethesda, MD, USA) to confirm successful surface functionalization and immobilization.

### 2.3. EDL-Gated BioFET Platform

A high-precision sensor chip was developed for the detection of TnI, a crucial biomarker for myocardial infarction. The sensing circuit device employed in this study is the BioFET Platform Model II, developed by STARX Co. in Taiwan. This chip incorporates gold electrodes embedded within a printed circuit board (PCB) and features a linear array of eight independently addressable sensors, with precise and high-throughput detection capabilities. Ultraviolet (UV) photolithography was employed to delineate the metal deposition areas. The fabrication process involved the sequential deposition of metal layers using electron beam evaporation, resulting in a multi-layered structure composed of a 200 Å titanium (Ti) adhesion layer, followed by a 500 Å gold (Au) layer, a 500 Å platinum (Pt) layer, and a final 2000 Å gold (Au) layer. Once the metal deposition was complete, a protective photoresist layer was applied to passivate the device. Subsequent photolithography steps were performed to expose two specific regions on the gold electrodes: one served as the sensing electrode connected to the gate terminal of a metal–oxide–semiconductor field-effect transistor (MOSFET), while the other functioned as a reference electrode for applying the gate bias.

To enhance the sensitivity of TnI detection, the sensor incorporates a planar electric double-layer structure with adjustable spacing. This design approach is inspired by previous research published in *Scientific Reports* (2018) [29], which indicated that at a fixed gate voltage, current gain diminishes as the spacing between the gate electrode and the FET channel increases, until reaching a saturation point. In this state, the characteristics of the electric double layer are determined solely by the gate voltage, rendering them independent of spatial variations, and thus aligning with conventional potentiometric theory. Moreover, operating within the linear regime—where decreasing the spacing enhances the current gain—enables sensitivities that surpass the theoretical Nernst limit, thereby significantly improving analytical performance.

In accordance with these principles, the sensing electrode was designed with dimensions of 600 μm × 600 μm, maintaining a gap of 65 μm between the two open regions to establish the planar electric double-layer spacing. This setup is depicted in Figure 1, which includes both a photograph of the extended gate wafer and a schematic representation of the measurement device. The device accommodates eight channels, allowing for simultaneous measurements, which helps to minimize experimental variability and enhance data reliability.

## 3. Results and Discussion

### 3.1. Immobilization Results

**Figure 2:**
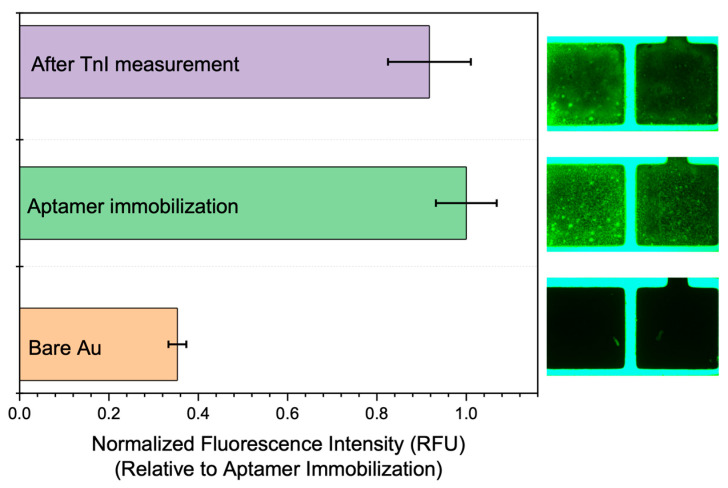
The fluorescence analysis data illustrate the progression of fluorescence intensity throughout the surface functionalization process. The signal starts with the cleaned bare gold (Au) surface, increases significantly after aptamer immobilization, and then decreases following the introduction of cardiac troponin I (TnI). All fluorescence values are normalized to the intensity after aptamer immobilization to reflect relative changes due to target binding.

The fluorescence analysis results presented in Figure 2 reveal a notable increase in fluorescence intensity following the immobilization of the Tro4 aptamer onto a bare gold surface. Notably, the fluorescence intensity after immobilization was two to three times greater than the initial measurements, thus confirming the successful attachment of the Tro4 aptamer to the gold surface.

A subsequent fluorescence analysis of the measured samples indicated a decrease in fluorescence intensity compared to the data recorded immediately after immobilization. This reduction suggests that the cardiac troponin I (cTnI) binding to the Tro4 aptamer was successful within the sensing device. The binding event likely induced conformational changes in the aptamer, which altered the orientation of the fluorescent molecules and led to the observed reduction in fluorescence intensity.

The Tro4 aptamer, selected through the SELEX (Systematic Evolution of Ligands by Exponential Enrichment) process, demonstrated remarkable binding affinity and specificity for cardiac troponin I (cTnI). It obtained a reported dissociation constant (Kd) of 270 pM, significantly lower than that of typical anti-cTnI antibodies, such as 20.8 nM. This indicates a stronger and more selective molecular interaction. Furthermore, Tro4 showed minimal cross-reactivity with structurally or functionally similar proteins, including cardiac troponin T (cTnT), troponin C (cTnC), human serum albumin (HSA), myoglobin, and B-type natriuretic peptide, which confirms its excellent molecular selectivity. This high specificity allows for the reliable differentiation of cTnI from potential interfering substances in complex biological environments, a crucial factor for accurate clinical diagnostics. For instance, fluorescence-based measurements in this study demonstrated distinct changes in signal intensity before and after the introduction of cTnI. These changes not only confirm the functional integrity of the aptamer on the sensing surface but also provide a direct indication of the binding event through fluorescence-quenching, serving as an additional verification of target capture.

Additionally, Tro4-based electrochemical aptasensors have shown high reproducibility, operational stability, and excellent performance in serum-based assays. Recent advancements in aptamer-functionalized nanostructures—such as tetrahedral DNA frameworks and gold nanoparticle-modified electrodes—have further improved capture efficiency and reduced nonspecific signals, making Tro4 an ideal recognition element in field-effect transistor (FET)- and fluorescence-based biosensor platforms.

Overall, these findings highlight the clinical potential of Tro4 aptamer-based biosensors for the sensitive and selective detection of cTnI, particularly in the early diagnosis of acute myocardial infarction [30,31,32].

### 3.2. Measurement

In this study, N-channel enhancement-mode metal–oxide–semiconductor field-effect transistors (E-MOS FETs) were utilized alongside extended-gate chips as the principal components of the biosensing platform. The extended-gate structure physically separates the sensing interface from the transistor body, enabling the chemical functionalization of the gate surface while protecting the semiconductor from direct exposure to aqueous environments. This characteristic is essential for biosensing applications that involve liquid-phase biological samples.

To assess the performance and operational stability of the MOSFETs in this platform, we conducted fundamental electrical characterizations, as depicted in Figure 1. The presented characterizations encompass the output characteristics, which delineate the relationship between drain current (I_d_) and drain voltage (V_d_) across various gate voltages (V_g_). Furthermore, the analysis includes the transfer characteristics of Id as a function of V_g_, along with the corresponding transconductance (g_m_) computed at a drain voltage of V_d_ = 2 V.

The results validate the linearity, gate control, and robustness of the device under the selected operating conditions, confirming its suitability as an amplification and readout unit in extended-gate field-effect transistor (EGFET)-based biosensors.

During the sensing measurements, troponin I (TnI) solutions of varying concentrations were carefully applied to the sensing electrode using a micropipette. A pulsed gate voltage, alternating between 0 V and 3.5 V with a pulse duration of 20 ms, was administered to the reference electrode while maintaining a constant drain-to-source voltage (V_d_) of 2 V. The drain current (I_d_) was continuously monitored throughout the process to observe interfacial charge variations induced through the binding of TnI molecules. A schematic illustration of the measurement configuration is also presented in Figure 1.

**Figure 3:**
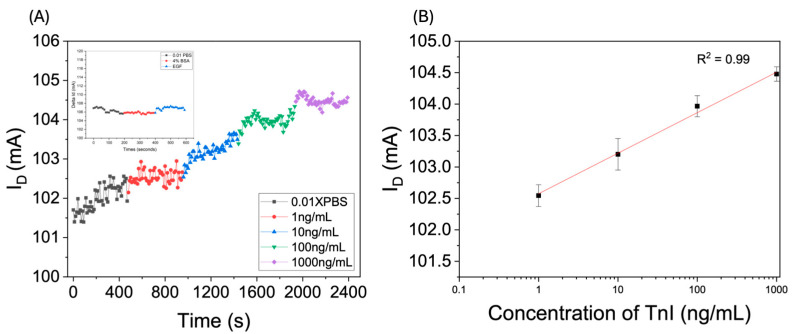
(**A**) Real-time variations in the drain current (I_d_) of the aptamer-based planar electric double-layer FET sensor in response to increasing concentrations of cardiac troponin I (TnI). The inset shows a specificity control experiment in which the sensing region was sequentially exposed to phosphate-buffered saline (PBS, without TnI), 4% bovine serum albumin (BSA), and epidermal growth factor (EGF), confirming that nonspecific biomolecules did not elicit significant current changes. (**B**) The calibration curve depicting the relationship between the change in drain current (ΔI_d_) and TnI concentration demonstrates a clear concentration-dependent response from the sensor.

The measurement results are illustrated in Figure 3, which demonstrates the time-dependent variations in the current induced by TnI solutions at varying concentrations. After stabilizing the chip in a phosphate-buffered saline (PBS) environment, a TnI solution with a concentration of 1 ng/mL was introduced at 480 s, resulting in a rapid increase in current. Following this, TnI solutions with concentrations of 10, 100, and 1000 ng/mL were added sequentially, with each producing a corresponding increase in the sensor current.

The relationship between the drain current and the logarithm of troponin I (TnI) concentration exhibits a clear linear correlation. This linear behavior arises from the inherent properties of troponin I, which has a basic isoelectric point (pI) of 9.3. In the weakly alkaline environment employed in this study, specifically at a pH of 7.4, the amino acid residues of TnI possess a net positive charge. This is due to decreased negative charges from acidic residues, such as glutamic and aspartic acids. In contrast, basic residues like lysine and arginine contribute additional positive charges. Consequently, TnI achieves a net positive charge.

When positively charged TnI binds to the aptamer in the sensing region, the electric double layer (EDL) formed in the dual-electrode area under the applied gate voltage undergoes polarization. The positive charges introduced by TnI enhance the positive charge density within the EDL, thereby increasing the positive potential shift (ΔVg) at the opposite end of the sensor. This mechanism underpins the observed sensitivity and linearity of the sensor’s response to varying TnI concentrations.

The enhanced sensitivity in the detection of troponin I (TnI) observed in this study is consistent with the principles of the electric double layer (EDL) and the Gouy–Chapman–Stern model. When an external voltage is applied at the electrode interface, it generates a structured double layer comprising two distinct regions: the Stern and diffuse layers. In the Stern layer, ions are directly adsorbed onto the electrode surface, creating a minimal potential gradient. Conversely, in the diffuse layer, the ion concentration gradually decreases with increasing distance from the surface, resulting in a spatially dependent charge distribution. The overall capacitance of the EDL is determined by the series combination of the Stern and diffuse layers, which can be expressed mathematically.

$$C_{St}=\frac{\epsilon_{0}\epsilon_{r,St}A}{l_{St}}$$

$$C_{diff}=\frac{\epsilon_{0}\epsilon_{r}A}{l_{diff}}$$

where $\epsilon_{0}\;$ is the permittivity of free space, $\epsilon_{r,St}$ represents the relative permittivity of the Stern layer, $l_{St}$ is the Stern layer thickness, A is the electrode surface area, $\epsilon_{r}$ is the relative permittivity of the diffuse layer, and $l_{diff}$ is approximately the Debye length. The total capacitance ($C_{EDL}$) of the EDL is governed by the series connection of these two layers:

$$\frac{1}{C_{EDL}}=\frac{1}{C_{St}}+\frac{1}{C_{diff}}$$

The surface potential ($\phi_{0}$) influences the EDL capacitance, with their interaction decribed by

$$\sigma_{EDL}=-\sigma_{0}=-C_{EDL}\cdot \phi_{0}$$

where $\sigma_{0}$ represents the surface charge and $\sigma_{EDL}$ represents the charge stored within the double layer. The relationship between surface charge density and surface potential is further defined using the Grahame equation:

$$\sigma_{EDL}=\sqrt{8c_{0}{\epsilon \epsilon }_{0}k_{B}T}\cdot \sinh\;(\frac{e\phi_{St}}{2k_{B}T})$$

Here, $c_{0}$ is the bulk electrolyte concentration, ϵ  denotes the relative permittivity of the buffer solution, $k_{B}$ is the Boltzmann constant, T represents the absolute temperature, e is the elementary charge, and $\phi_{St}$ is the electrostatic potential at the Stern plane. These theoretical formulations provide a fundamental understanding of how the EDL modulates interfacial capacitance and charge distribution, ultimately contributing to the enhanced sensitivity observed in the proposed TnI detection platform.

**Figure 4:**
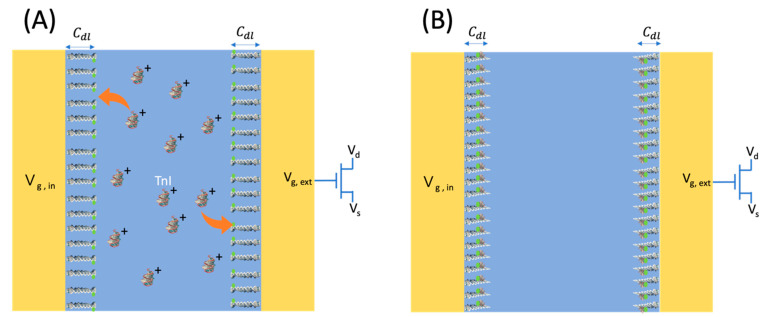
(**A**) Schematic diagram of the Aptamer-Based Planar Electric Double-Layer FET sensor in its unbound state with TnI. The red arrows indicate the binding interaction between TnI molecules and surface-immobilized aptamers. (**B**) Schematic diagram of the Aptamer-Based Planar Electric Double-Layer FET sensor upon binding with TnI.

When positively charged troponin I (TnI) proteins are adsorbed onto the electrode through aptamer binding, the electric double layer (EDL) structure is modified, resulting in detectable changes in capacitance due to voltage fluctuations. As illustrated in Figure 4, the planar electrode configuration creates a strong localized electric field that promotes rapid charge accumulation within the EDL, thereby enhancing the capacitance changes and improving detection sensitivity. Furthermore, the increased surface area of the electrode amplifies interactions with the analyte, further enhancing detection sensitivity.

The change in gate voltage (ΔVg) can be effectively modeled using the parallel plate capacitor model, which establishes a relationship between the change in surface charge density (Δσ), the effective separation between charge layers (d), and the permittivity of the medium (ϵ):

$$\Delta Vg=\frac{\left(\Delta \sigma \right)d}{\epsilon }$$

According to the Gouy–Chapman theory, the surface charge density exhibits a proportional increase with the concentration of biomolecules, such as troponin I, upon the binding of aptamers. Consequently, variations in gate bias establish a linear relationship between the drain-source current and the concentration of troponin I.

As depicted in Figure 3B, the sensor demonstrates a high sensitivity of $21,390\;\mu A\cdot {cm}^{-2}/decade$, with an estimated detection limit (LOD) of 0.0001467 decade. The LOD was determined using the following standard equation:

$$LOD=\frac{3.3\sigma }{S}$$

where S represents the sensor sensitivity and σ is the standard deviation corresponding to the lowest measured concentration. The calculated LOD of 0.0001467 decade corresponds to an approximate ionic concentration $2.51\times 10^{16}\;ions/m^{3}$. This corresponds to a relative concentration change of approximately 1.00034-fold, indicating the high sensitivity of the sensor, even to extremely small variations in analyte concentration.

To further assess the electrostatic interactions governing detection, the Debye length ($\lambda_{D}$) was calculated using the following expression:

$$\lambda_{D}=\sqrt{\frac{\varepsilon k_{B}T}{nq^{2}}}$$

where $k_{B}$ is the Boltzmann constant, T denotes the absolute temperature, n represents the ion concentration, and q is the elementary charge. Given that deionized water has a relative permittivity ($\varepsilon_{r}$) of 80, and that a 0.02× phosphate-buffered saline (PBS) solution was used in this study—with an estimated ionic strength of approximately 3 mM—the corresponding ion concentration was calculated as $n\approx 1.81\times 10^{24}\;ions/m^{3}$. When substituted into the equation, the Debye length was estimated to be approximately 7.93 μm.

The Tro4 aptamer employed in this study consists of 30 nucleotides, resulting in a molecular length of approximately 10.2 nm. This design suggests that the planar electric double-layer structure effectively transmits the applied electric field across the sensing interface, thereby alleviating the charge-screening effects that typically limit detection sensitivity. These findings highlight the effectiveness of the proposed sensor architecture in facilitating precise and ultra-sensitive biomolecular detection.

Moreover, Table 1 presents a summary of several recent Troponin I detection technologies, detailing the detection methods, analytical techniques, sensitivity or linear ranges, and limits of detection (LOD), along with corresponding references. This comparison clearly demonstrates the competitive performance of our proposed aptamer-based planar electric double-layer FET sensor in relation to current state-of-the-art approaches. Notably, our sensor achieves an LOD of 10 pg/mL and a sensitivity of 7.46 × 10^9^ μA·mM⁻^1^·cm⁻^2^, positioning it as a strong candidate for ultra-sensitive biomarker detection, particularly in low-concentration environments.

## 4. Conclusions

This study introduces a groundbreaking aptamer-based, planar electric, double-layer field-effect transistor (FET) system that enables the highly sensitive and specific detection of troponin I (TnI). This cutting-edge sensing platform effectively addresses the limitations of conventional ion-sensitive detectors, which are restricted by Debye length effects, thanks to its unique planar electric double-layer design, which significantly enhances detection performance. By integrating TnI-specific aptamers within this advanced double-layer configuration, our sensor achieves an impressive detection limit of 0.0001467 decade. It displays a robust linear response across a clinically relevant range of 1 ng/mL to 100 ng/mL.

Moreover, the use of interchangeable extended gate chips greatly improves the modularity and scalability of the system, making it ideally suited for practical applications in home healthcare. This advancement not only broadens the potential for effective home healthcare solutions but also signifies a notable progression in biomarker detection. Additionally, it supports real-time patient monitoring and diagnostics in non-clinical settings. These innovations underscore the transformative potential of this sensing technology for point-of-care testing and personalized medicine, paving the way for more accessible and effective healthcare solutions.

## Figures and Tables

**Table 1:** Comparison of various sensing methods and materials for Troponin I (TnI) detection, including detection limits (LOD), linear detection ranges, and references. The present work demonstrates an extended-gate FET sensor with a Tro4 aptamer-modified gate achieving superior sensitivity.

Sensing Method	Material/Nanoparticle	Structure	LOD	Linear Range	Reference
Colorimetric	AuNPs	Ab and BSA conjugated Anti-BSA	0.01 ng/mL	0.10–1.42 ng/mL	[33]
Colorimetric	AuNPs	Antibody/AuNPs-biotin-dsDNA-AuNP as amplifier	1 ng/mL	1–1000 ng/mL	[34]
Colorimetric	AuNPs	Ab conjugated AuNPs	10 pg/mL	30.0–1000 ng/mL	[35]
Colorimetric	AuNPs–shell hybrid nanobeads	Ab conjugated	9.9 pg/mL	N.M.	[36]
Colorimetric	L-CGNPs	Ab–CSNPs–Ab–CS-GNPs	0.129 ng/mL	0.01–2.0 ng/mL	[37]
Fluorescence	CdSe/ZnS QD	Ab conjugated	0.049 pg/mL	0.04–50.0 ng/mL	[38]
SERS	ZrMOF@AuNPs	Gold nanorods	5.6 × 10⁻^3^ ng/mL	0.08–200 ng/mL	[39]
Luminescence	UCNP	Ab conjugated with MB	0.01 ng/mL	128.4–190.6 ng/mL	[40]
Electrochemical	AuNPs	AuNP-polydopamine conjugated	0.015 ng/mL	0.01–500 ng/mL	[41]
Electrochemical	Mesoporous silica NP	Ab conjugated with HRP	5.6 pg/mL	0.01–6.0 ng/mL	[42]
Electrochemical	AuNPs	Magnetic beads (MB)	0.84 pg/mL	30–1000 ng/mL	[43]
Electrochemical FET (This work)	Extended-gate FET with Tro4 aptamer	FET with aptamer-modified gate	0.0001467 decade	10–10^4^ pg/mL	This Work

“N.M.” indicates “Not Mentioned” in the original reference.

## Data Availability

The data supporting the reported results in this study are available upon reasonable request from the corresponding author.
